# Biodegradable Persistent Luminescence Nanoparticles as Pyroptosis Inducer for High‐Efficiency Tumor Immunotherapy

**DOI:** 10.1002/advs.202406340

**Published:** 2024-08-19

**Authors:** Lin Liu, Junpeng Shi, Jinyuan Wang, Linping He, Yan Gao, Peng Lin, Yutong Han, Ping'an Ma, Jun Lin, Yun Zhang

**Affiliations:** ^1^ State Key Laboratory of Structural Chemistry Fujian Institute of Research on the Structure of Matter Chinese Academy of Sciences Fuzhou 350002 China; ^2^ Xiamen Key Laboratory of Rare Earth Photoelectric Functional Materials Xiamen Institute of Rare Earth Materials Haixi Institute Chinese Academy of Sciences Xiamen 361021 China; ^3^ University of Chinese Academy of Sciences Beijing 100049 China; ^4^ Australian Institute for Bioengineering and Nanotechnology The University of Queensland St Lucia QLD 4067 Australia; ^5^ State Key Laboratory of Rare Earth Resource Utilization Changchun Institute of Applied Chemistry Chinese Academy of Sciences Changchun 130022 China

**Keywords:** biodegradable, immunotherapy, persistent luminescence nanoparticles, pyroptosis

## Abstract

Pyroptosis possesses potent antitumor immune activity, making pyroptosis inducer development a promising direction for tumor immunotherapy. Persistent luminescence nanoparticles (PLNPs) are highly sensitive optical probes extensively employed in tumor diagnosis and therapy. However, a pyroptosis inducer based on PLNPs has not been reported yet. Herein, polyethylene glycol–poly lactic acid‐co‐glycolic acid (PEG–PLGA: PP) modified biodegradable CaS:Eu^2+^ (CSE@PP) PLNPs are synthesized as a pyroptosis inducer for tumor immunotherapy for the first time. The synthesized CSE@PP possesses biowindow persistent luminescence (PersL) and pH‐responsive degradation properties, allowing it to remain stable under neutral pH but degrade when exposed to weak acid (pH < 6.5). During degradation within the tumor, CSE@PP constantly releases H_2_S and Ca^2+^ while its PersL gradually fades away. Thus, the PersL signal can self‐monitor H_2_S and Ca^2+^ release. Furthermore, the released H_2_S and Ca^2+^ result in mitochondrial dysfunction and the inactivation of reactive oxygen species scavenging enzymes, synergistic facilitating intracellular oxidative stress, which induces caspase‐1/GSDM‐D dependent pyroptosis and subsequent antitumor immune responses. In a word, it is confirmed that CSE@PP can self‐monitor H_2_S and Ca^2+^ release and pyroptosis‐mediated tumor Immunotherapy. This work will facilitate biomedical applications of PLNPs and inspire pyroptosis‐induced tumor immunotherapy.

## Graphical abstract

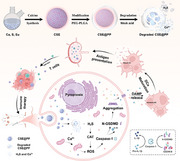

## 1 Introduction

Immunotherapy can achieve a new breakthrough in oncotherapy due to its high specificity, efficiency, and capacity to minimize body damage.^[^ ^1^ ^]^ However, its therapeutic effect on various malignant tumors is severely restricted by the nonimmunogenic tumor microenvironment (TME).^[^ ^2^ ^]^ Pyroptosis, a newly discovered mode of programmed cell death, is usually characterized by DNA fragmentation, cell swelling and bubbling, and the release of cellular proinflammatory contents, which is triggered by the inflammatory caspase‐mediated gasdermin cleavage.^[^ ^3^ ^]^ Studies have shown that pyroptosis can induce effective antitumor immune responses.^[^ ^4^ ^]^ The inflammatory danger signals released by tumor cells during pyroptosis can recruit immune cells to the TME and promote their functions, such as enhancing the phagocytosis of tumor cells by tumor‐associated macrophages, as well as the number and functions of tumor‐infiltrating natural‐killer cells and CD8^+^ T lymphocytes. Recently, pyroptosis inducer development to trigger tumor cell pyroptosis for enhancing tumor immunotherapy has become a hotspot.^[^ ^5^ ^]^

Nanoparticles exhibit unique advantages in inducing pyroptosis and tumor immunotherapy because of their high tumor enrichment efficiency, loading capacity, and low biotoxicity.^[^ ^6^ ^]^ Researchers have developed pyroptosis inducers with various functions to trigger tumor cell pyroptosis and activate antitumor immune activity.^[^ ^7^ ^]^ Ion interference therapy, in particular, has garnered significant attention due to its advantages of in vivo metabolism and free external activation.^[^ ^8^ ^]^ This therapy focuses on designing degradable nanoparticles that, when degraded in tumor cells, release large amounts of ions such as Ca^2+^, Na^+^, K^+^, Zr^4+^, or Zn^2+^, leading to intracellular ion overload, homeostasis imbalances, and subsequent pyroptosis.^[^ ^9^ ^]^ Despite remarkable advancements in biodegradable nanoparticle research, the inability to monitor the release of ions within the tumor hampers the treatment process. Additionally, the efficiency of pyroptosis induced by ion overload alone still needs improvement.

Persistent luminescence nanoparticles (PLNPs) are bioluminescent probes widely used in diagnosis and therapy in recent years.^[^ ^10^ ^]^ PLNPs can generate persistent luminescence (PersL) after excitation, characterized by the separation of excitation and emission, which can completely eliminate the interference of biological autofluorescence, making them extremely sensitive in biological imaging.^[^ ^11^ ^]^ Therefore, PLNPs have innate advantages in monitoring ion release and guiding therapy. However, currently developed PLNPs for diagnostic and therapeutic applications have stable structures that do not allow for in vivo degradation and ion release, resulting in their inability to induce pyroptosis and severely limiting PLNP application in tumor immunotherapy.^[^ ^12^ ^]^

**Scheme 1:**
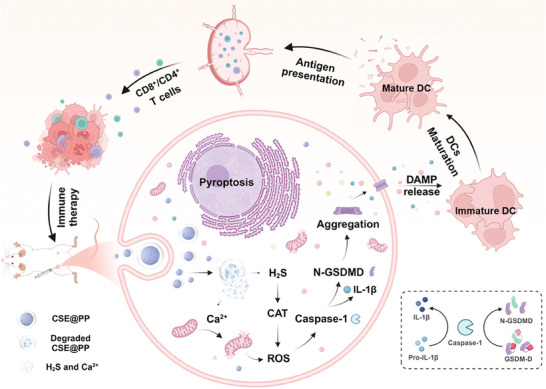
Mechanism of CSE@PP inducing pyroptosis for tumor immunotherapy.

Herein, we reported for the first time a polyethylene glycol–poly lactic acid‐co‐glycolic acid (PEG–PLGA: PP) modified biodegradable CaS:Eu^2+^ (CSE@PP) PLNPs and found that CSE@PP could induce tumor cell pyroptosis and subsequent anti‐tumor immune response. The synthesized CaS:Eu^2+^ (CSE) possesses biowindow PersL and pH‐responsive degradation properties. In the tumor, CSE@PP can be degraded to release H_2_S and Ca^2+^ gradually, while its PersL gradually weakens until disappears. On the one hand, the fading PersL can be used to monitor the release of H_2_S and Ca^2+^ during treatment. On the other hand, H_2_S and Ca^2+^ release lead to a surge in intracellular reactive oxygen species (ROS) by inducing mitochondrial dysfunction and inactivation of ROS‐scavenging enzyme, and further activate caspase‐1/GSDM‐D dependent pyroptosis (**Scheme** **1**). Finally, the antitumor immunity activity of CSE@PP was demonstrated via a 4T1‐bearing tumor mice model.

## 2 Results and Discussion

**Figure 1:**
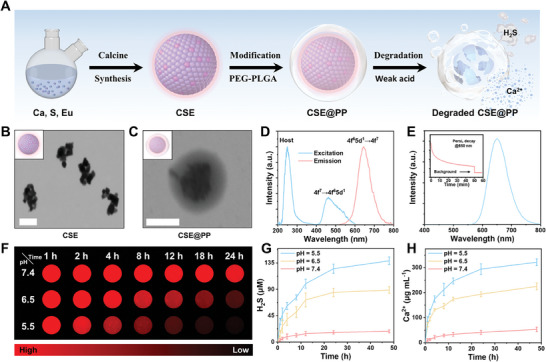
A) Schematic representation for the preparation process and degradation property of CSE@PP. TEM images of B) CSE (scale bar: 100 nm) and C) CSE@PP (scale bar: 100 nm). D) Normalized excitation and emission spectra of CSE@PP. E) PersL emission spectrum and PersL decay curve of CSE@PP. F) CCD imaging of CSE@PP under different pH conditions. Release of G) H_2_S (*n* = 3 in each group; mean ± SD) and H) Ca^2+^ of CSE@PP under different pH conditions (*n* = 3 in each group; mean ± SD).

**Figure** **1**A shows the preparation process and degradation properties of CSE@PP. First, CSE was prepared via combined high‐temperature coprecipitation and calcination methods.^[^ ^13^ ^]^ After calcination, the synthesized CSE formed agglomerates with a particle size of ≈100 nm (Figure 1B) and a cubic crystal structure (Figure S1, Supporting Information). To improve biocompatibility^[^ ^14^ ^]^ CSE was coated with PP to obtain CSE@PP (Figure 1C; Figure S2, Supporting Information). The successful preparation of CSE@PP was confirmed through ultraviolet (UV)–visible (vis)–near‐infrared (NIR) absorption spectra (Figure S3, Supporting Information) and Fourier transform infrared spectra (Figure S4, Supporting Information). Meanwhile, the stability of CSE@PP was confirmed by dynamic light scattering and zeta potential analysis (Figures S5 and S6, Supporting Information). Figure 1D shows the optical performance of CSE@PP. Under 254 nm excitation, a band peaked at 650 nm was observed, which is attributed to the 4f^6^5d^1^→4f^7^ transition of Eu^2+^.^[^ ^15^ ^]^ Furthermore, monitored at 650 nm, two bands peaked at 254 and 480 nm, attributed to the intrinsic defect and 4f^7^→4f^6^5d^1^ transition of Eu^2+^.^[^ ^15^ ^]^ After white light excitation, CSE@PP exhibited PersL emission at 650 nm (Figure 1E), and the PersL located in the biowindow is of great advantage for in vivo imaging.^[^ ^16^ ^]^ Additionally, the PersL of CSE@PP can be repeatedly excited by white light after decay, indicating the PersL excitation stability of CSE@PP (Figure S7, Supporting Information).

Next, the degradation properties of CSE@PP were investigated. No significant change was observed in the PersL signal of CSE@PP under the neutral pH condition (pH = 7.4), while under weak acid conditions (pH = 6.5 and 5.5), the PersL signal gradually weakened (Figure 1F), indicating the destruction of its crystal structure under weak acid conditions. The release of H_2_S (Figure 1G) and Ca^2+^ (Figure 1H) further confirmed the degradation of CSE@PP. In addition, the release content of H_2_S and Ca^2+^ varied at different pH levels and was negatively correlated with the PersL signal. Therefore, this signal can be used to monitor H_2_S and Ca^2+^ release. These results indicate that CSE@PP is a pH‐activated degradable nanoparticle with self‐monitoring capability for the follow‐up study of its intracellular behavior.

**Figure 2:**
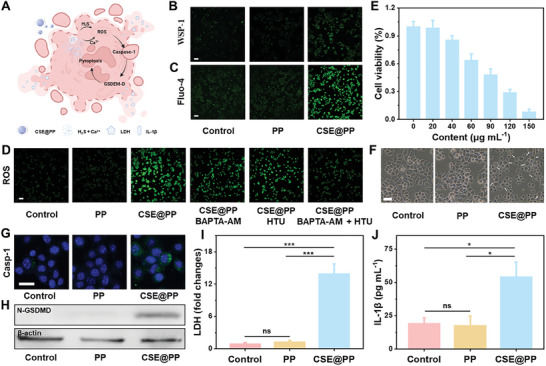
A) Schematic representation of the pyroptosis induced by H_2_S and Ca^2+^ ions released from CSE@PP. Intracellular release of B) H_2_S (scale bar: 200 µm) and C) Ca^2+^ from CSE@PP (scale bar: 200 µm). D) Intracellular ROS production under different treatment conditions (scale bar: 200 µm). E) Cytotoxicity assay of 4T1 cells treated with different concentrations of CSE@PP (*n* = 5 in each group; mean ± SD). F) Morphology of 4T1 cells under different treatment conditions (scale bar: 30 µm). G) Immunofluorescence images of activated caspase‐1 after 4T1 cells under different treatment conditions (scale bar: 50 µm). H) Western blotting analysis of GSDM‐D after 4T1 cells under different treatment conditions. Release of I) LDH (*n* = 5 in each group; mean ± SD, ^***^ *p* < 0.001) and J) IL‐1β of 4T1 cells under different treatment conditions (*n* = 5 in each group; mean ± SD, ^*^ *p* < 0.05).

**Figure** **2**A shows CSE@PP triggering 4T1 cell pyroptosis. CSE@PP enters tumor cells and is degraded, releasing H_2_S and Ca^2+^, which synergistically increase intracellular oxidative stress levels and subsequent caspase‐1/GSDM‐D‐dependent pyroptosis. First, CSE@PP cell uptake was investigated by labeling it with Rhodamine B. The red fluorescence from rhodamine B in 4T1 cells confirmed the effective endocytosis of CSE@PP (Figure S8, Supporting Information). Then, the endocytic CSE@PP was degraded to release H_2_S and Ca^2+^ ions, detected by the fluorescence probes WSP‐1 and fluo‐4 acetoxymethyl ester (AM), respectively (Figure 2B,C). Ion overload and a surge in H_2_S can induce intracellular oxidative stress,^[^ ^17^ ^]^ thereby assessing the ROS level in the 4T1 cells treated with CSE@PP by the 2′,7′‐dichlorodihydrofluorescein diacetate (DCFH‐DA) probe. The 4T1 cells treated with CSE@PP exhibited stronger green fluorescence than the control and PP groups, indicating CSE@PP‐induced intracellular oxidative stress. To distinguish the role of H_2_S and Ca^2+^ in ROS generation, the Ca^2+^ chelator BAPTA‐AM and the H_2_S scavenger hypotaurine (HTU) were employed. Compared with the CSE@PP group, the addition of BAPTA‐AM or HTU reduced green fluorescence (Figure 2D), indicating that H_2_S and Ca^2+^ promote intracellular oxidative stress, respectively. Moreover, adding both BAPTA‐AM and HTU significantly decreased green fluorescence (Figure 2D), indicating that H_2_S and Ca^2+^ have a synergistic effect on ROS generation.

Although CSE@PP has been verified to induce ROS by releasing H_2_S and Ca^2+^ ions, the underlying mechanism requires further investigation. The significant rise in ion concentration within cells could profoundly impact cellular functions, particularly leading to mitochondrial dysfunction and an increase in ROS.^[^ ^17b^ ^]^ Mitochondrial membrane potential (ΔΨm), an important index for evaluating the normal function of mitochondria, was measured by the 5,5′,6,6′‐tetrachloro‐1,1′,3,3′‐tetraethyl‐benzimidazolylcarbocyanine iodide (JC‐1) probe, which can emit red fluorescence at normal ΔΨm and green fluorescence when ΔΨm collapses. As shown in Figure S9 (Supporting Information), ΔΨm was depolarized in 4T1 cells treated with CSE@PP. The results confirmed that CSE@PP caused mitochondrial dysfunction. Intracellular redox homeostasis is modulated by ROS‐scavenging enzymes in the TME, such as catalase (CAT). H_2_S has been reported to suppress CAT activity, thereby increasing ROS production.^[^ ^18^ ^]^ Therefore, the CAT activity was measured using corresponding assay kits. Compared with the control and PP groups, the CAT activity was suppressed in 4T1 cells treated with CSE@PP (Figure S10, Supporting Information). These results confirm that the H_2_S and Ca^2+^ release leads to a surge in intracellular reactive oxygen species (ROS) by inducing mitochondrial dysfunction and inactivation of CAT.

The cytotoxicity of CSE@PP was then evaluated using the Cell Counting Kit‐8 (CCK‐8) assay kit (Figure 2E). The results showed that CSE@PP could effectively inhibit cell proliferation in a dose‐dependent manner. When the concentration of CSE@PP increased to 150 µg mL^−1^, ≈10% of 4T1 cells were alive, indicating the cell‐killing ability of the former. The cytotoxicity was also confirmed through calcein‐AM/PI staining (Figure S11, Supporting Information). Furthermore, numerous cells with large bubbles, a characteristic of pyroptosis in the CSE@PP group (Figure 2F), implied that CSE@PP killed 4T1 cells via pyroptosis. According to previous reports, increased ROS production could induce caspase‐1/GSDM‐D‐dependent pyroptosis.^[^ ^19^ ^]^ Thus, caspase‐1 expression examined through immunofluorescence images (Figure 2G) showed an increase of activated caspase‐1 (green fluorescence) in the CSE@PP group. This can lead to the cleavage gasdermin‐D (GSDM‐D), generating N‐GSDMD and eliciting pyroptosis. As shown in Figure 2H, the N‐GSDMD was upregulated in the CSE@PP group, indicating that CSE@PP activated the caspase‐1/GSDMD‐dependent pyroptosis pathway. Additionally, N‐GSDMD can perforate cells to release contents by oligomerization and binding to cell membranes,^[^ ^20^ ^]^ In the CSE@PP group, extracellular lactic dehydrogenase (LDH) levels increased significantly (Figure 2I). In addition, interleukin‐1β (IL‐1β), a marker released during pyroptosis,^[^ ^21^ ^]^ was also higher than the other two groups (Figure 2J). These results confirm that CSE@PP is a strong pyroptosis inducer.

**Figure 3:**
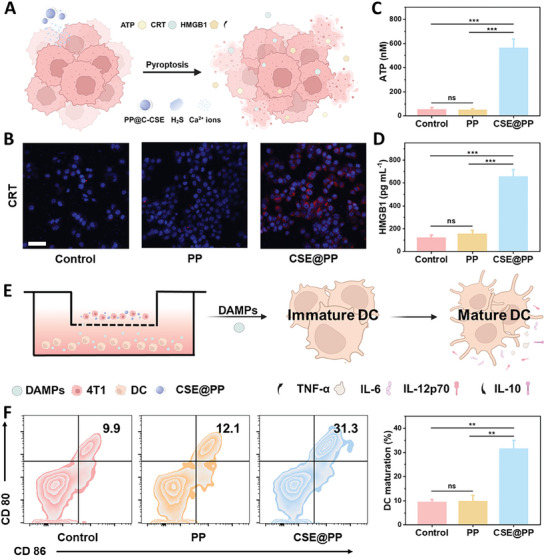
A) Schematic representation for DAMPs released from 4T1 cells treated with CSE@PP. B) Immunofluorescence analysis of CRT (red fluorescence) in 4T1 cells under different treatment conditions (scale bar: 100 nm). Release of C) ATP (*n* = 5 in each group; mean ± SD, ^***^ *p* < 0.001) and D) HMGB1 of 4T1 cells under different treatment conditions (*n* = 5 in each group; mean ± SD, ^***^ *p* < 0.001). E) Schematic representation for DC maturation‐induced 4T1 cells under different treatment conditions. F) Flow cytometry analysis of DC maturation induced 4T1 cells under different treatment conditions (*n* = 5 in each group; mean ± SD, ^**^ *p* < 0.01).

The secretion of inflammatory molecules and the release of cellular contents during pyroptosis have been confirmed to activate robust antitumor immunity.^[^ ^4a^ ^]^ Immunogenicity enhancement was characterized by measuring damage‐associated molecular patterns (DAMPs), including nuclear high‐mobility group box 1 protein (HMGB1), calreticulin (CRT), and adenosine triphosphate (ATP).^[^ ^22^ ^]^ These DAMPs can trigger antigen presentation and antitumor immune responses. Thus, we detect CRT exposure, as well as ATP and HMGB1 release in 4T1 cells treated with CSE@PP (**Figure** **3**A). As shown in Figure 3B, the red fluorescence in the CSE@PP group is significantly enhanced, confirming CRT exposure. Compared to the control and PP groups, the cells treated with CSE@PP released a large number of ATP (Figure 3C) and HMGB1 (Figure 3D). The activation of dendritic cells (DC), one of the most important antigen‐presenting cells, is related to cytokine secretion, and their maturation is stimulated by the release of DAMPs (Figure 3E), further activating immune responses.^[^ ^23^ ^]^ As shown in Figure 3F, CSE@PP treatment significantly increased the maturation of DC. Moreover, cytokine secretion related to DC activation was also evaluated through an enzyme‐linked immune sorbent assay (ELISA). As shown in Figures S12A–D (Supporting Information), the secretion of IL‐12, tumor necrosis factor α (TNF‐α), and IL‐6 was significantly increased, while IL‐10 was decreased, suggesting the potential of CSE@PP to promote immune activation.

**Figure 4:**
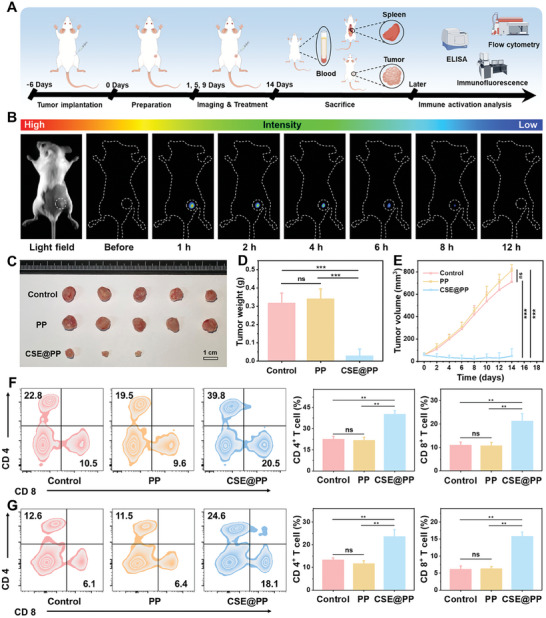
A) Schematic representation of the imaging and treatment process in mice with unilateral tumors. B) PersL images of 4T1 bearing tumor mice. C) Photographs of unilateral tumors under different treatment conditions. D) Average weights of tumors under different treatment conditions (*n* = 5 in each group; mean ± SD, ^***^ *p* < 0.001). E) Tumor growth curves under different treatment conditions (*n* = 5 in each group; mean ± SD, ^***^ *p* < 0.001). Flow cytometry analysis of F) spleen (*n* = 5 in each group; mean ± SD, ^**^ *p* < 0.01) and G) tumor tissue of 4T1 tumor‐bearing mice under different treatment conditions (*n* = 5 in each group; mean ± SD, ^**^ *p* < 0.01).

We then evaluated the antitumor performance of CSE@PP in vivo. BALB/c mice were inoculated with 4T1 cells, followed by therapy and immunoassay (**Figure** **4**A). First, we monitored CSE@PP degradation after intratumoral injection (Figure 4B and Figure S13, Supporting Information). Compared with subcutaneous injection, the PersL signal was found and decreased continuously until disappeared by intratumor injection, indicating that CSE@PP can be degraded inside the tumor and release H_2_S and Ca^2+^. Due to this release, the tumors of mice treated with CSE@PP were significantly suppressed, and two tumors were completely cured (Figure 4C–E). The hematoxylin and eosin (H&E) staining of tumor slides also confirmed the efficient antitumor effect (Figure S14, Supporting Information). In addition, mice weight (Figure S15, Supporting Information) and major organs (Figure S16, Supporting Information) were not influenced after treatment, suggesting the biocompatibility and biosafety of CSE@PP.

Next, the immunoassay was performed through flow cytometry analysis. First, DC maturation in tumor‐draining lymph nodes was evaluated. As shown in Figure S17 (Supporting Information), the DC maturation level was significantly increased in the CSE@PP group. Mature DC can promote proliferation and intratumoral infiltration of T lymphocytes.^[^ ^24^ ^]^ Therefore, the amount of CD4^+^ T and CD8^+^ T cells in the spleen and tumor tissue was measured by flow cytometry and immunofluorescence analysis. The CD4^+^ T and CD8^+^ T cells were significantly increased in the spleen (Figure 4F; Figure S18, Supporting Information) and tumor tissue (Figure 4G; Figure S19, Supporting Information). Moreover, TNF‐α, IL‐6, IFN‐γ, and IL‐12 levels were significantly increased in mice treated with CSE@PP (Figure S20A–D, Supporting Information). These results indicated that CSE@PP induced immune activity in mice to suppress tumor growth.

**Figure 5:**
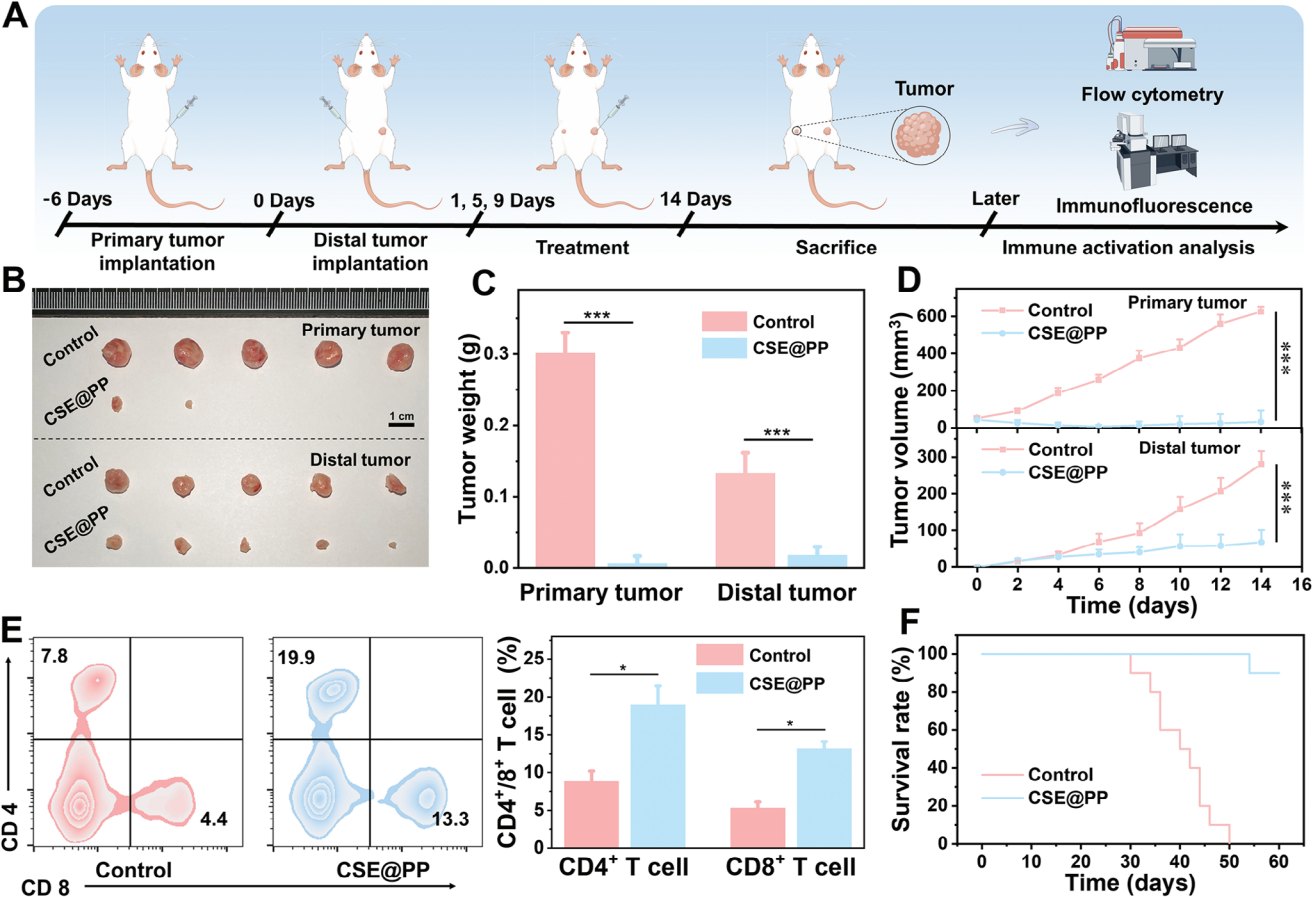
A) Schematic representation of the treatment process in mice with bilateral tumors. B) Photographs of primary and distal tumors under different treatment conditions. C) Average weights of primary and distal tumors under different treatment conditions (*n* = 5 in each group; mean ± SD, ^***^ *p* < 0.001). D) Primary and distal tumor growth curves under different treatment conditions (*n* = 5 in each group; mean ± SD, ^***^ *p* < 0.001). E) Flow cytometry analysis of distal tumor of 4T1 tumor‐bearing mice under different treatment conditions (*n* = 5 in each group; mean ± SD, ^*^ *p* < 0.05). F) Mice survival curve.

To further verify CSE@PP‐induced immune activation, a bilateral 4T1 tumor model was established in vivo (**Figure** **5**A). After treating the right back tumor, the primary and distal tumor growth was suppressed (Figure 5B–D). The results suggest that CSE@PP can activate the immune system of mice to achieve an abscopal effect while treating the primary tumor. To explore the relationship between distal tumor suppression and immune activation, the distal tumor and corresponding lymph nodes were extracted for immunoassay. As shown in Figure S21 (Supporting Information), mature DC in distal tumor‐draining lymph nodes was increased, and the increased intratumoral infiltration of CD4^+^ T and CD8^+^ T cells was found in the distal tumor (Figure 5E and S22, Supporting Information). Furthermore, mice survival was monitored after tumor inoculation, and the treated mice survived significantly longer compared with the control group (Figure 5F), indicating that CSE@PP can achieve tumor immunotherapy and improve mice survival.

## 3 Conclusion

This work reported in vivo degradable PLNPs for the first time and demonstrated their ability to induce tumor cell pyroptosis and activate antitumor immunity. As a Ca^2+^ and H_2_S reservoir, CSE@PP has a pH‐responsive degradation capacity, leading to an increase in intracellular ROS production by disrupting ΔΨm and inhibiting CAT activity, thereby activating the caspase‐1/GSDMD‐dependent pyroptosis pathway. During degradation, the PersL signal can be used to monitor Ca^2+^ ions and H_2_S release. Finally, the antitumor activity of CSE@PP was demonstrated via the 4T1 tumor‐bearing mice model. This study greatly expands PLNP application in immunotherapy and provides new insights into designing PLNP‐based pyroptosis inducers.

## 4 Experimental Section

### Chemicals and Materials

Ca(CH_3_COO)_2_ (99.9%), Eu(CH_3_COO)_2_ (99.99%), N,N′‐diphenylthiourea (DPTU), cyclohexane, oleylamine (OAm), trioctylamine (TOA), and tetrahydrofuran were purchased from Aladdin (China). Oleic acid (OA) was purchased from Sigma–Aldrich (America). HTU was purchased from Maokang Biotechnology (China). WSP‐1 was purchased from GlpBio (America). BAPTA‐AM was obtained from Invitrogen (America). The HMGB1 assay kit was purchased from Ruixin Biotechnology (China). PLGA–PEG was purchased from Ruixi Biotech Biochemistry Technology (China). ROS assay kit, LDH assay kit, ATP assay kit, bicinchoninic acid assay kit, CCK‐8 assay kit, calcein‐AM/propidium iodide assay kit, bicinchoninic acid (BCA) protein assay kit, enhanced chemiluminescence (ECL) kit, 2′,7′‐dichlorofluorescein diacetate, JC‐1, fluo‐4 AM, and antiCRT were acquired from Beyotime (China). H_2_S assay kit was acquired from Solarbio (China). Anticaspase‐1 and antiN‐GSDMD were purchased from Abcam (America). IL‐1β, IL‐6, IL‐10, IL‐12p70, TNF‐α, and INF‐γ assay kits were purchased from Neobioscience (China). Antimouse CD4‐APC, antimouse CD8‐PE, antimouse CD3‐FITC, antimouse CD11‐FITC, antimouse CD86‐APC, antimouse CD80‐PE, GM‐CSF, and IL‐4 were purchased from PeproTech (America).

### Synthesis of CSE@PP

CSE was synthesized via a combined high‐temperature coprecipitation and calcination method. Then, 1 mmol Ca(CH_3_COO)_2_ and 0.005 mmol Eu(CH_3_COO)_2_ were mixed with 2 mL of OA, 6 mL of TOA, and 12 mL of OAm in a three‐necked round‐bottom flask. The resulting solution was heated to 150 °C under N_2_ flow to remove the residual water and oxygen and then stirred to form a clear solution. After cooling down to room temperature, 10 mL of an ethanol solution containing 3 mmol of DPTU was added, and the solution was stirred at 80 °C to remove the ethanol. Then, the resulting solution was heated to 320 °C under N_2_ flow for 90 min and then cooled down to room temperature. The obtained nanoparticles were collected after adding ethanol. To improve the optical performance, the as‐synthesized nanoparticles were annealed at 900 °C for 1.5 h in a furnace under H_2_/N_2_ (5%/95%) reducing atmosphere. Then, the nanoparticles were thoroughly ground and ultrasonicated for 0.5 h. After that, the suspension was centrifuged at 2000 rpm for 5 min to precipitate the large and aggregated CSE. The supernatant was collected and dried. To improve the biocompatibility, 50 mg CSE and 300 mg PLGA–PEG (PP) were added to 5 mL tetrahydrofuran, and the resulting solution was sonicated for 10 min. Then, the solution was injected into 5 mL of deionized water under vigorous stirring for 5 min at room temperature. Finally, the solution was centrifuged at 14 000 rpm for 30 min to obtain CSE@PP.

### Characterization

X‐ray diffraction patterns were measured through a Miniflex 600 X‐ray diffractometer (Rigaku, Japan) with a Cu Kα X‐ray tube operated at 45 kV and 40 mA. The FLS920 fluorescence spectrometer (Edinburgh, UK) was used to record the excitation and emission spectra, PersL spectrum, and decay curve. UV–vis–NIR absorption spectra were recorded using a Cary 5000 spectrophotometer (Agilent, USA). Zetasizer Lab (malvern, UK) was used to analyze dynamic light scattering and zeta potential. Transmission electron microscopy images were acquired using the H‐7650 system (Hitachi, Japan). Elemental content for degrading CSE@PP was determined using ULTIMA 2 inductively coupled plasma optical emission spectrometer (HORIBA, French). Fourier transform infrared spectroscopy was performed using a Nicolet iS50 FT‐IR spectrometer (Thermo Fisher, USA). The Ca^2+^ content for the degradation of CSE@PP was determined using an inductively coupled plasma optical emission spectrometer (ICP‐OES, ULTIMA 2, HORIBA Scientific Co., Ltd., French). PersL images were obtained through the IVIS Lumina II imaging system (Caliper, USA). Confocal laser scanning microscopy (CLSM) images were obtained through A1‐MP CLSM (Nikon, Japan).

### Cellular Uptake Assay

The 4T1 cells were seeded in 12‐well plates and incubated with 1 mL of CSE@PP (100 µg mL^−1^) at different times. Then, they were washed several times with phosphate‐buffered saline (PBS) and fixed with 4% paraformaldehyde for 15 min. After washing with PBS, the 4T1 cells were stained by 4′‐6‐diamidino‐2‐phenylindole (DAPI) for CLSM imaging.

### Intracellular H_2_S, Ca^2+^, ROS, and ΔΨm Assays

After incubation of the 4T1 cells with 1 mL CSE@PP (100 µg mL^−1^), the medium was replaced, and H_2_S, Ca^2+^, ROS, and ΔΨm probes (WSP‐1, fluo‐4 AM, DCFH‐DA, and JC‐1, respectively) were added. Then, these cells were washed several times with PBS for CLSM imaging.

### Cytotoxicity Assay

The 4T1 cells were seeded in 96‐well plates and incubated overnight with different concentrations of CSE@PP for 24 h. Then, the medium was washed several times with PBS, and a serum‐free medium (100 µL) containing CCK‐8 solution (10 µL) was added to each well. The adsorptions of the medium solution were recorded at 450 nm using a microplate reader.

### Live/Dead Cell Staining

After incubation of the 4T1 cells with 1 mL CSE@PP (100 µg mL^−1^), the medium was replaced and the calcein‐AM/PI stain was added. Then, they were washed several times with PBS for CLSM imaging.

### Cellular Morphology Assay

After incubation of the 4T1 cells with 100 µL CSE@PP (100 µg mL^−1^) and washing with PBS, their images were obtained through a fluorescence microscope.

### Extracellular ATP, HMGB1, LDH, and IL‐1β Assays

The 4T1 cells seeded in six‐well plates were incubated with 2.5 mL CSE@PP (150 µg mL^−1^). Then, the medium was centrifugated to collect the supernatant, which was used to measure ATP, HMGB1, LDH, and IL‐1β levels via corresponding testing kits.

### Caspase‐1 and CRT Assays

After incubation of the 4T1 cells with 1 mL CSE@PP (100 µg mL^−1^), they were washed with PBS, fixed with 4% paraformaldehyde, and submerged in an immunostaining blocking solution at 4 °C overnight. Then, the solution was removed and incubated with caspase‐1 or CRT antibodies at 4 °C overnight. After washing the 4T1 cells, Alexa Fluor 488‐conjugated goat antirabbit IgG was added. Then, DAPI was added for nuclear staining. Finally, the 4T1 cells were washed several times with PBS for CLSM imaging.

### Western Blotting

The treated 4T1 cells were harvested, washed with PBS, and lysed in an ice–water bath in radioimmunoprecipitation assay buffer. The cells were then centrifuged at 4 °C to collect the supernatant, and the protein content was measured using a BCA protein assay kit. A certain amount of 5× loading buffer was added as an indicator and boiled at 90 °C for 8 min. The protein was separated and then transferred to a polyvinylidene fluoride membrane. The primary antibody was carried out overnight at 4 °C, and the secondary antibodies were carried out for 1 h at 24 °C. Finally, they were washed several times using a washing buffer, and then their signals were detected using an ECL kit.

### In Vitro DC Maturation Assay

Immature DCs were extracted from BALB/c mice and cultured in a complete RPMI 1640 medium containing IL‐4 (10 ng mL^−1^) and GM‐CSF (20 ng mL^−1^). The DC maturation experiment proceeded through a transwell assay. The 4T1 cells and immature DCs were seeded in a six‐transwell chamber and six‐well plates. Then, the 4T1 cells were incubated with 2.5 mL of CSE@PP (150 µg mL^−1^). After incubation, the immature DCs were collected and washed with PBS for flow cytometric analysis after staining with antiCD80, antiCD86, and antiCD11. Furthermore, the supernatant was used to measure IL‐12, IL‐10, IL‐6, and TNF‐α via corresponding ELISA kits.

### In Vivo Antitumor Experiments

All animal experiments involving animals were conducted in accordance with the guidelines of the National Regulation of China for the Care and Use of Laboratory Animals and were approved by the Animal Ethics Committee of Fujian Medical University (IACUC FJMU 2024‐0053). For the unilateral subcutaneous tumor model, the 4T1 cells resuspended in RPMI 1640 medium were injected into the right back of BALB/c mice. When the tumor volume reached ≈30–50 mm^3^, the mice were randomly divided into three groups treated with PBS, PP, and CSE@PP (200 µg) for further experiments. For the bilateral subcutaneous tumor model, the 4T1 cells were subcutaneously seeded into the right back of BALB/c mice when the primary tumors were treated. All reagents were injected intratumorally at tumor sites on days 1, 5, and 9, and body weight and tumor volume were monitored every 2 days. Tumor volume was calculated as L × W^2^/2. On the 14th day, all mice were sacrificed, and the major organs, tumor, spleen, lymph nodes, and serum were collected for analysis. In addition, the tumors from different individuals at the same time were photographed by transverse arrangement.

The lymph nodes were used to assess DC maturation (antiCD80, antiCD86, and antiCD11) through flow cytometry. The tumor and spleen were also used to assess the infiltration and proliferation of T lymphocytes (antiCD3, antiCD4, and antiCD8) through flow cytometry and immunohistochemical methods. Finally, the major organs were stained with H&E, and the serum was used to measure cytokines (TNF‐α, IL‐6, IFN‐γ, and IL‐12).

### In Vivo PersL Imaging

When the tumor volume reached ≈30–50 mm^3^, CSE@PP was injected into the established tumor. After 5 min of white light excitation, the 4T1 tumor–bearing mice were anesthetized and imaged at different times using the IVIS Lumina II imaging system. In addition, the controlled experiment was performed using normal mice by subcutaneous injection.

### Statistical Analysis

The experimental results were expressed as mean ± SD. Differences between different treating groups were evaluated by one sample T‐test in OriginPro 2018, and ^*^ *p* < 0.05, ^**^ *p* < 0.01, and ^***^ *p* < 0.001 were regarded as statistically significant.

## Conflict of Interest

The authors declare no conflict of interest.

## Supporting information

Supporting Information

## Data Availability

Research data are not shared.
