# Supplementary material for: Biodegradable Persistent Luminescence Nanoparticles as Pyroptosis Inducer for High‐Efficiency Tumor Immunotherapy
Source: Adv Sci (Weinh). 2024 Aug 19;11(39):2406340. doi: 10.1002/advs.202406340 (PMC11497027; doi:10.1002/advs.202406340)
Supplement: Supplementary file 1 — Supporting Information [file ADVS-11-2406340-s001.docx]

Supporting Information

**Biodegradable Persistent Luminescence Nanoparticles as Pyroptosis Inducer for High-Efficiency Tumor Immunotherapy**

Lin Liu*^abd^*, Junpeng Shi*^abd^**, Jinyuan Wang*^ab^*, Linping He*^ab^*, Yan Gao*^ab^*, Peng Lin*^ab^*, Yutong Han*^e^*, Ping’an Ma*^cd^*, Jun Lin*^cd^**, and Yun Zhang*^abd^**

^a^State Key Laboratory of Structural Chemistry, Fujian Institute of Research on the Structure of Matter, Chinese Academy of Sciences, Fuzhou 350002, China

^b^Xiamen Key Laboratory of Rare Earth Photoelectric Functional Materials, Xiamen Institute of Rare Earth Materials, Haixi Institute, Chinese Academy of Sciences, Xiamen 361021, China

^c^State Key Laboratory of Rare Earth Resource Utilization Changchun Institute of Applied Chemistry, Chinese Academy of Sciences, Changchun, 130022, China

^d^University of Chinese Academy of Sciences, Beijing 100049, China

^e^Australian Institute for Bioengineering and Nanotechnology, The University of Queensland, St Lucia, QLD, 4067, Australia

*Corresponding author: [shijunpeng10@mails.ucas.edu.cn](mailto:shijunpeng10@mails.ucas.edu.cn) (J. Shi), [jlin@ciac.ac.cn](mailto:jlin@ciac.ac.cn) (J. Lin), [zhangy@fjirsm.ac.cn](mailto:zhangy@fjirsm.ac.cn) (Y. Zhang)

Keywords: persistent luminescence nanoparticles, pyroptosis, immunotherapy, biodegradable


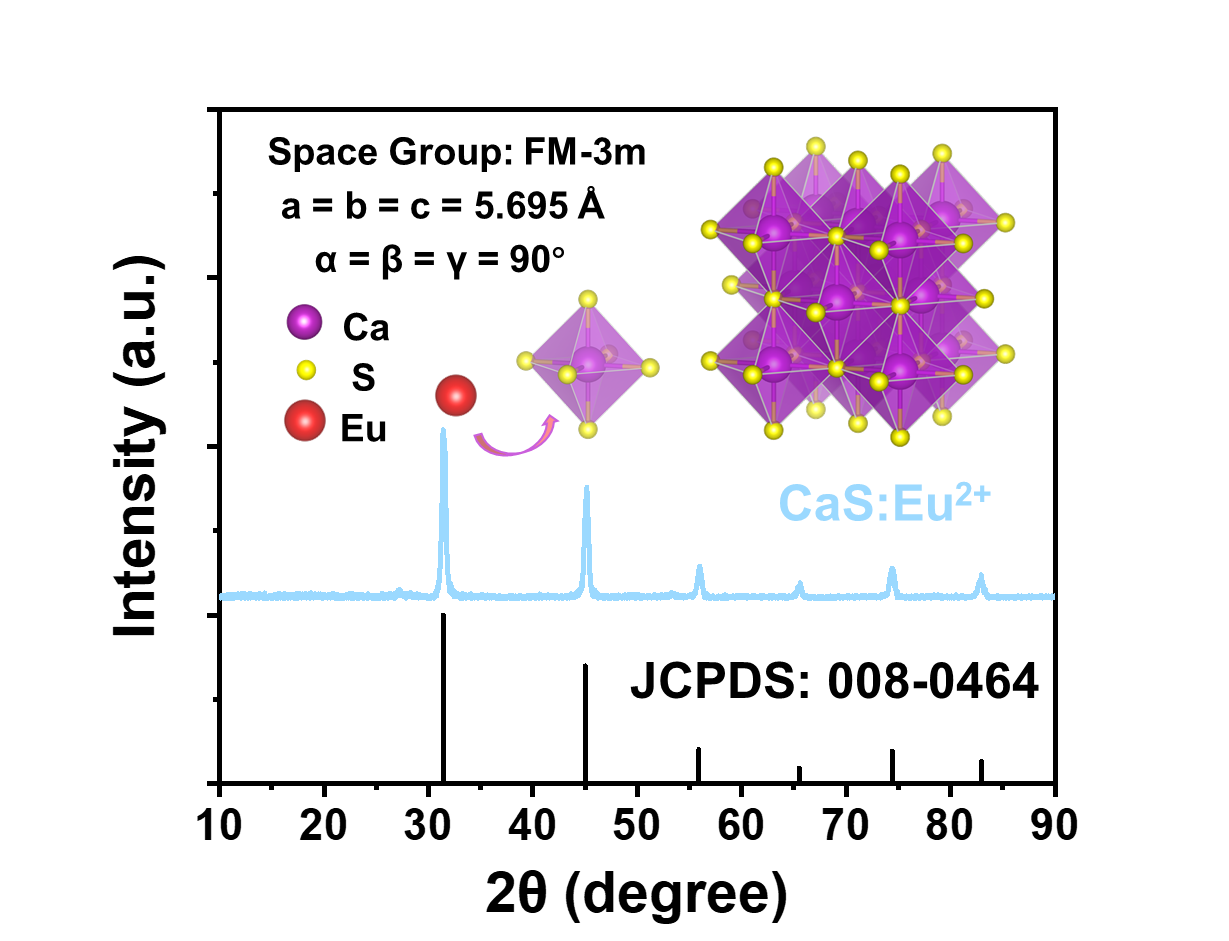


**Figure S1**. XRD pattern of CSE and its crystal structure.


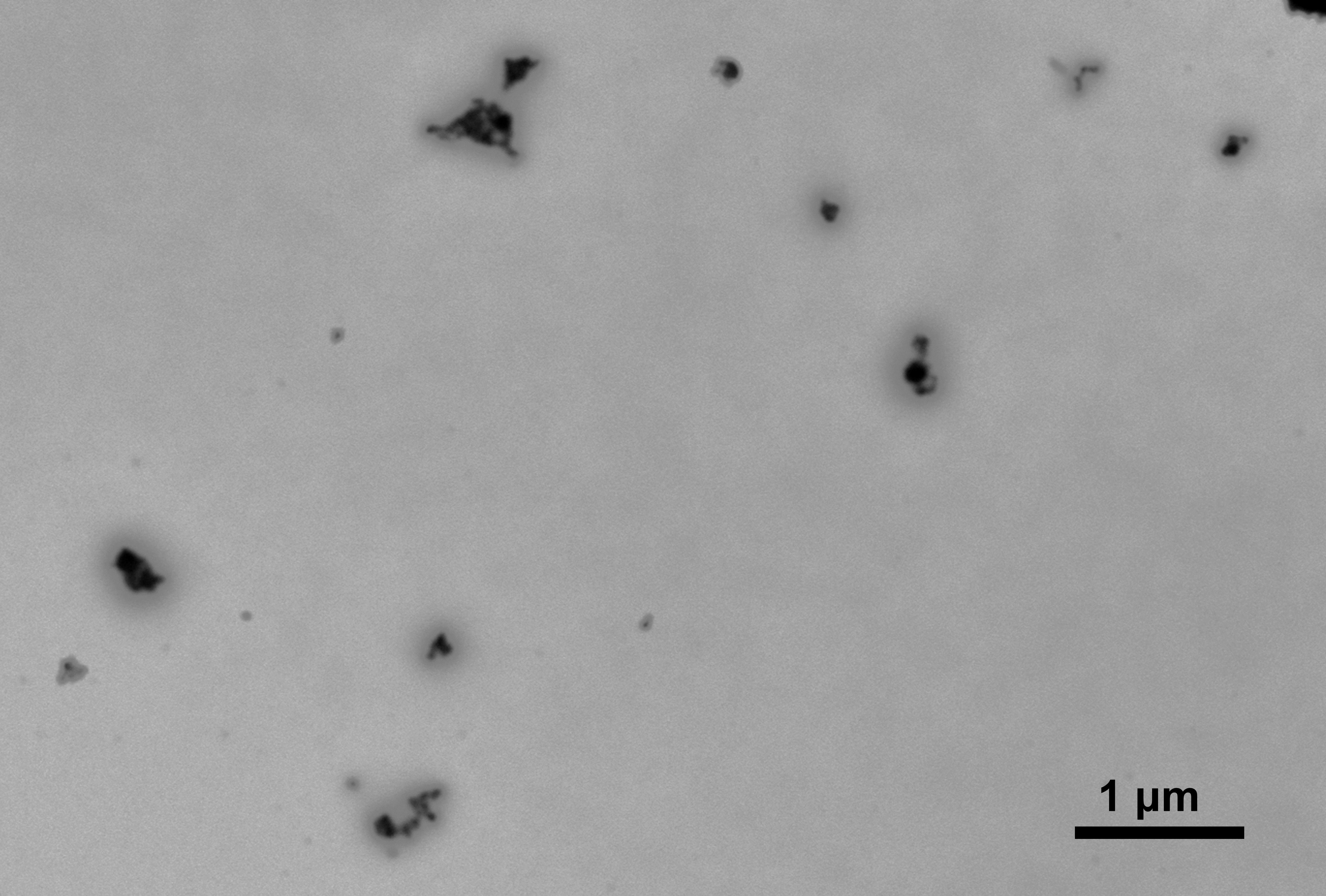


**Figure S2**. TEM image of CSE@PP.


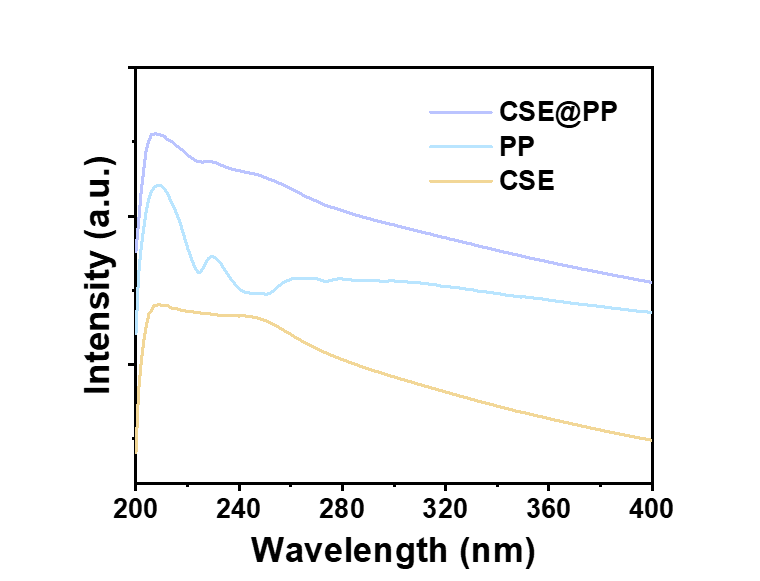


**Figure S3**. UV–vis–NIR absorption spectra of CSE, PP, and CSE@PP.


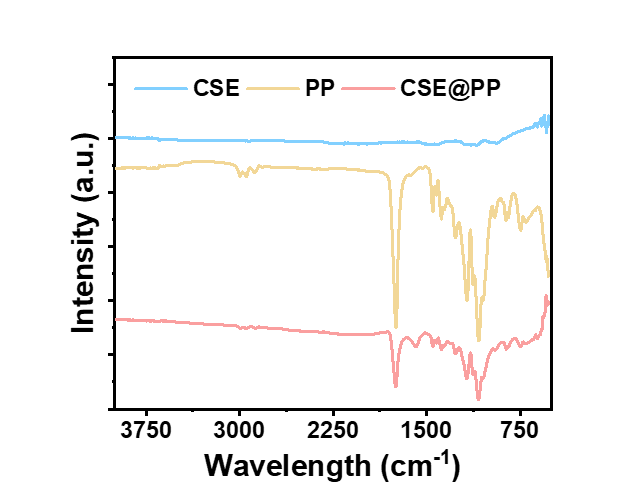


**Figure S4**. Fourier transform infrared spectra of CSE, PP, and CSE@PP.


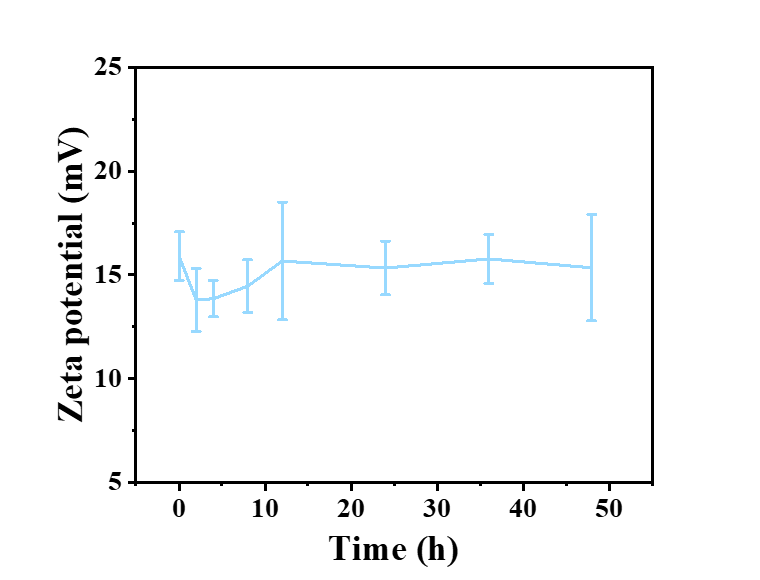


**Figure S5.** Zeta potential of CSE@PP in deionized water over 48 h (n = 3 in each group; mean ± SD).


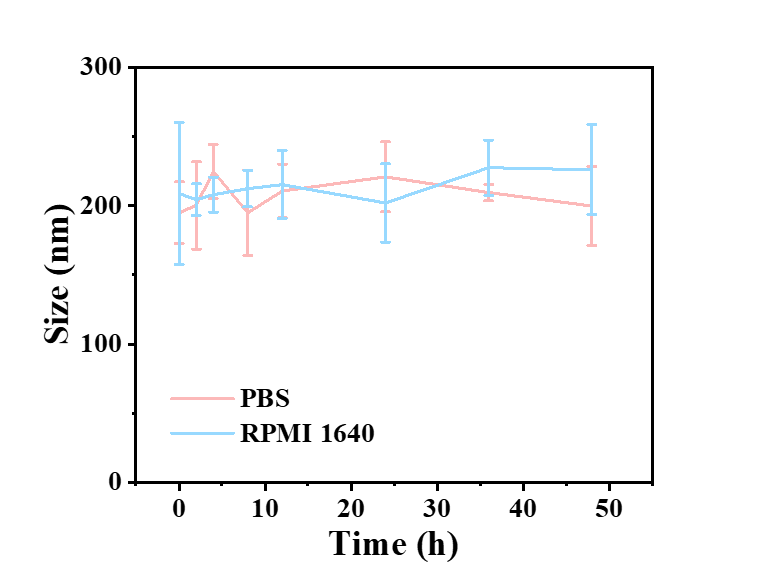


**Figure S6**. Dynamic light scattering of CSE@PP in PBS or RPMI 1640 medium over 48 h. Data are presented as the mean ± SD (n = 3 in each group; mean ± SD).


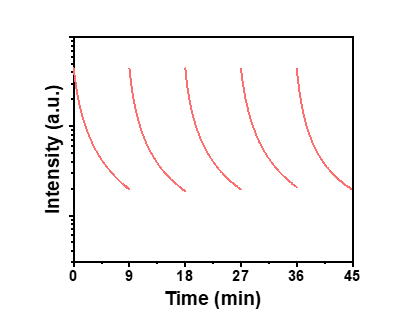


**Figure S7**. PersL decay curve of CSE@PP after repeated white light excitation, where the PersL decay trends and intensity are consistent, confirming its optical stability.


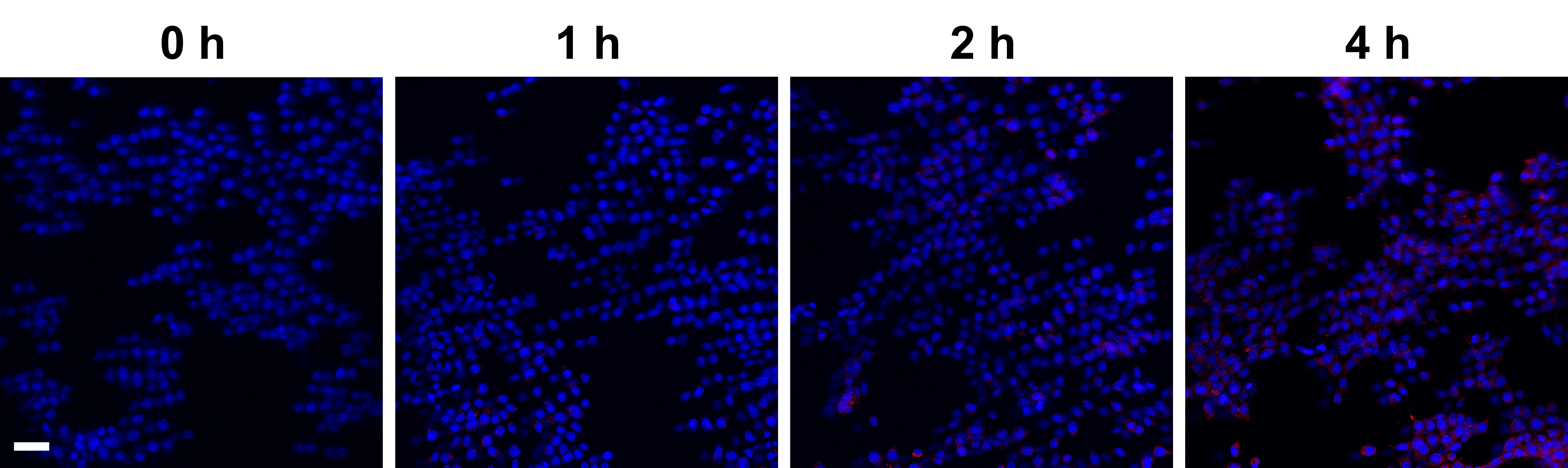


**Figure S8**. Cell uptake of CSE@PP (scale bar: 100 μm).


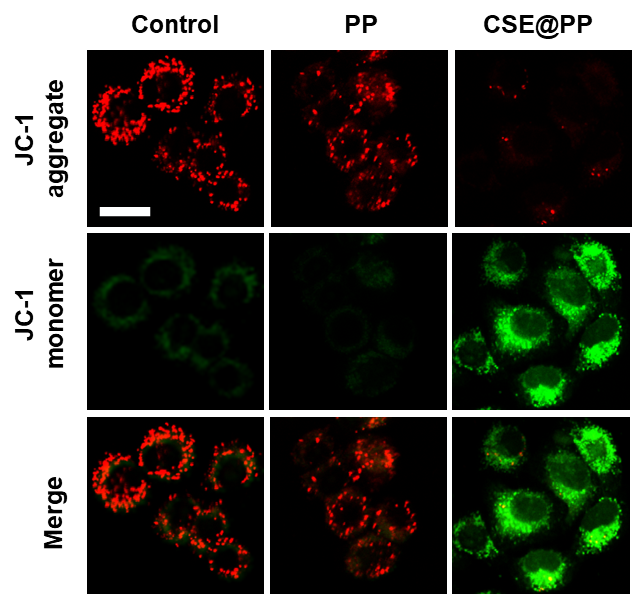


**Figure S9**. Mitochondrial membrane potential (ΔΨm) under different treatment conditions (scale bar: 20 μm).


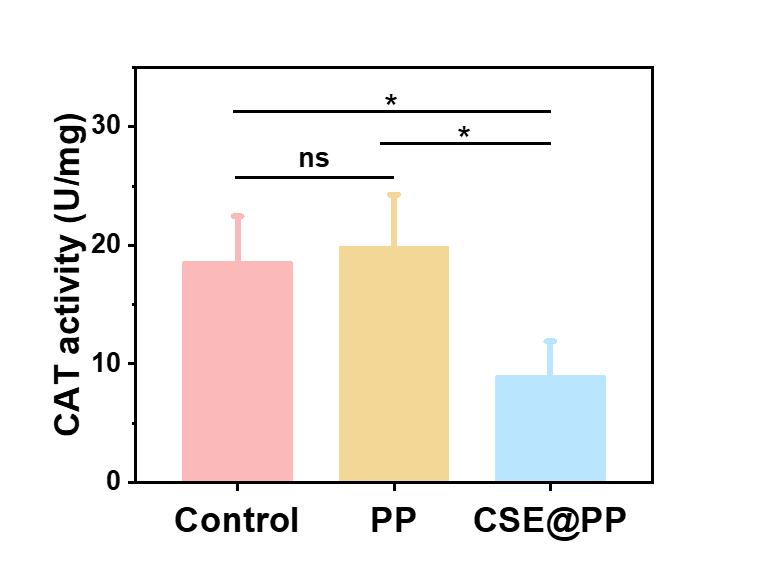


**Figure S10**. CAT activity of 4T1 cells under different treatment conditions (n = 5 in each group; mean ± SD, *p < 0.05).


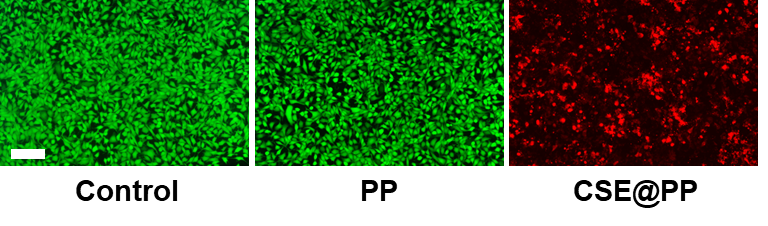


**Figure S11**. Calcein-AM/PI staining of 4T1 cells under different treatment conditions (scale bar: 100 μm).


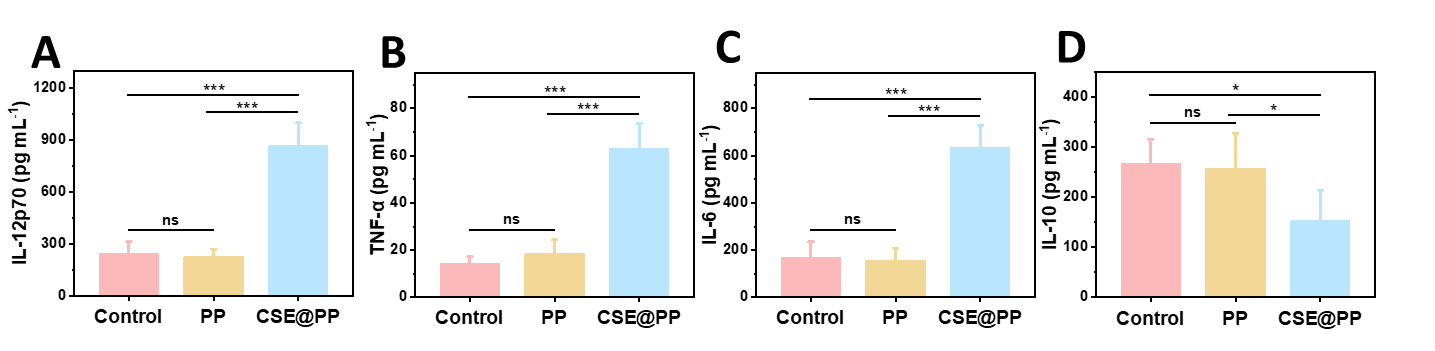


**Figure S12**. Secretion of cytokines related to DCs activation. (A) IL-12, (B) TNF-α, (C) IL-6, and (D) IL-10. (n = 5 in each group; mean ± SD, *p < 0.05, ***p < 0.001)


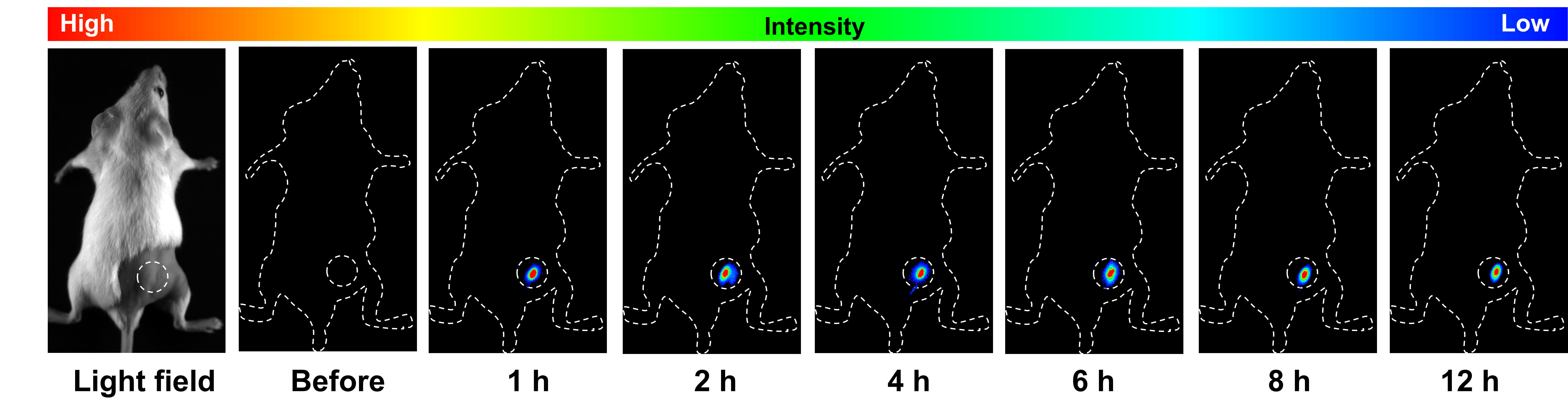


**Figure S13**. PersL images of normal mice by subcutaneous injection of CSE@PP.


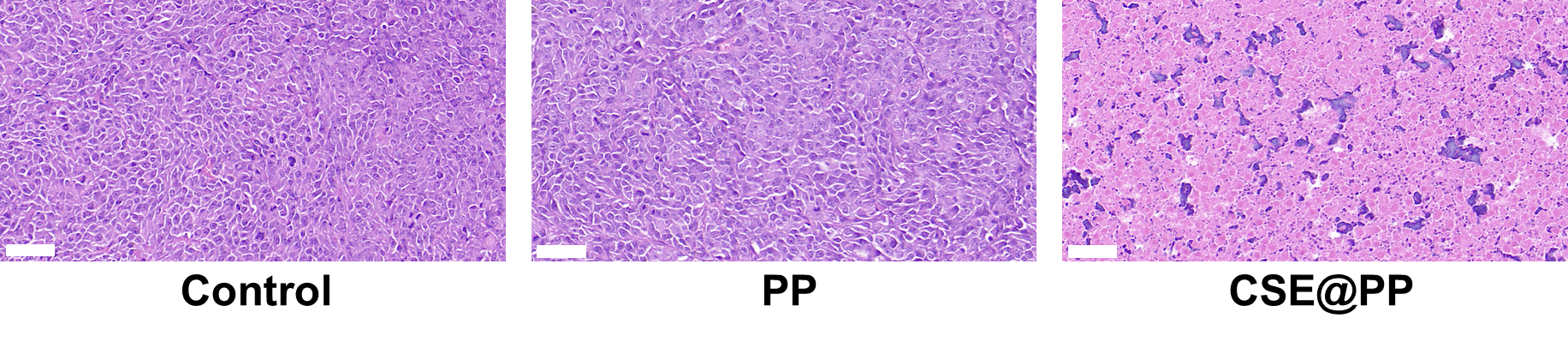


**Figure S14**. H&E staining in tumor tissues (scale bar: 100 μm).


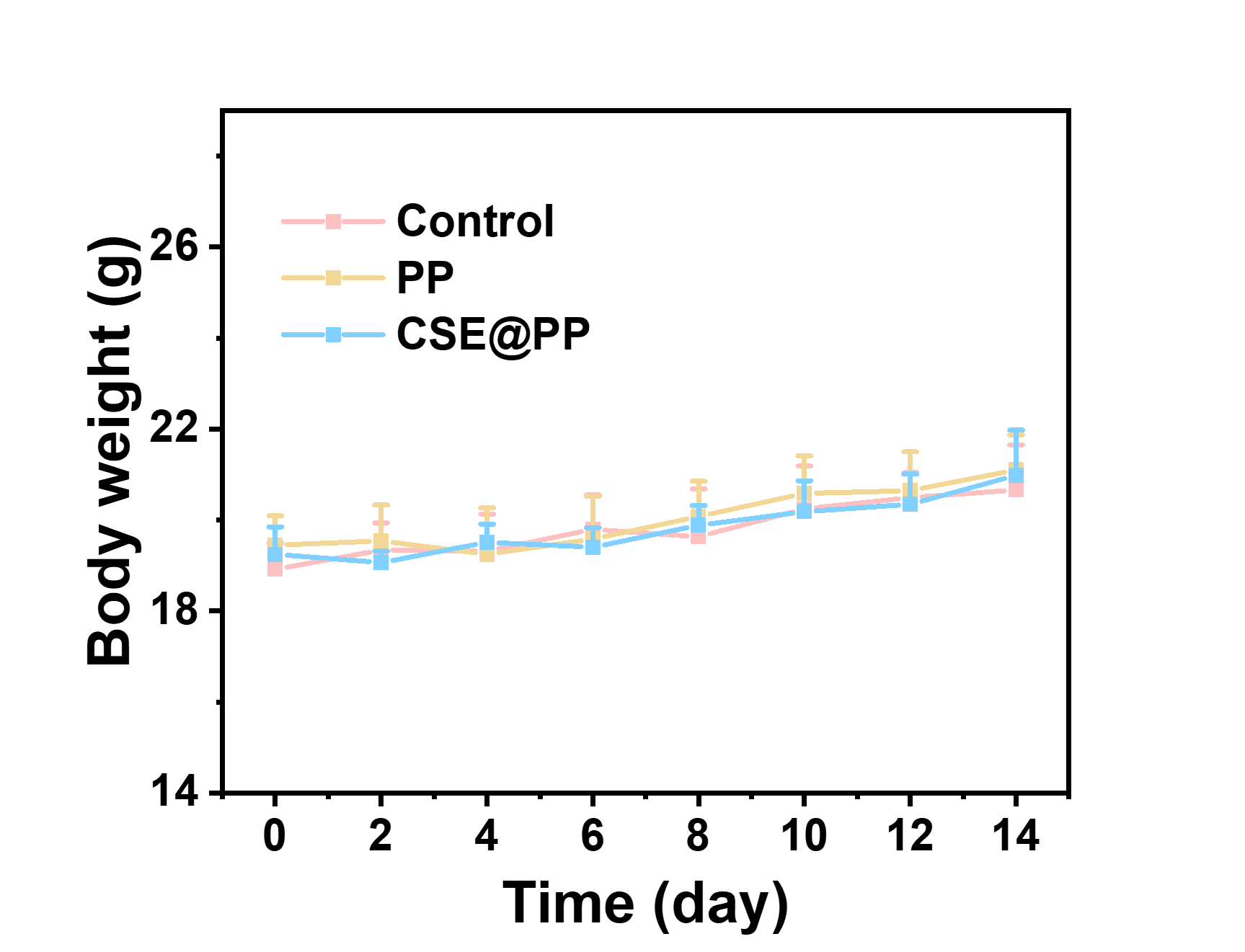


**Figure S15**. Change in weight of mice during treatment (n = 5 in each group; mean ± SD).


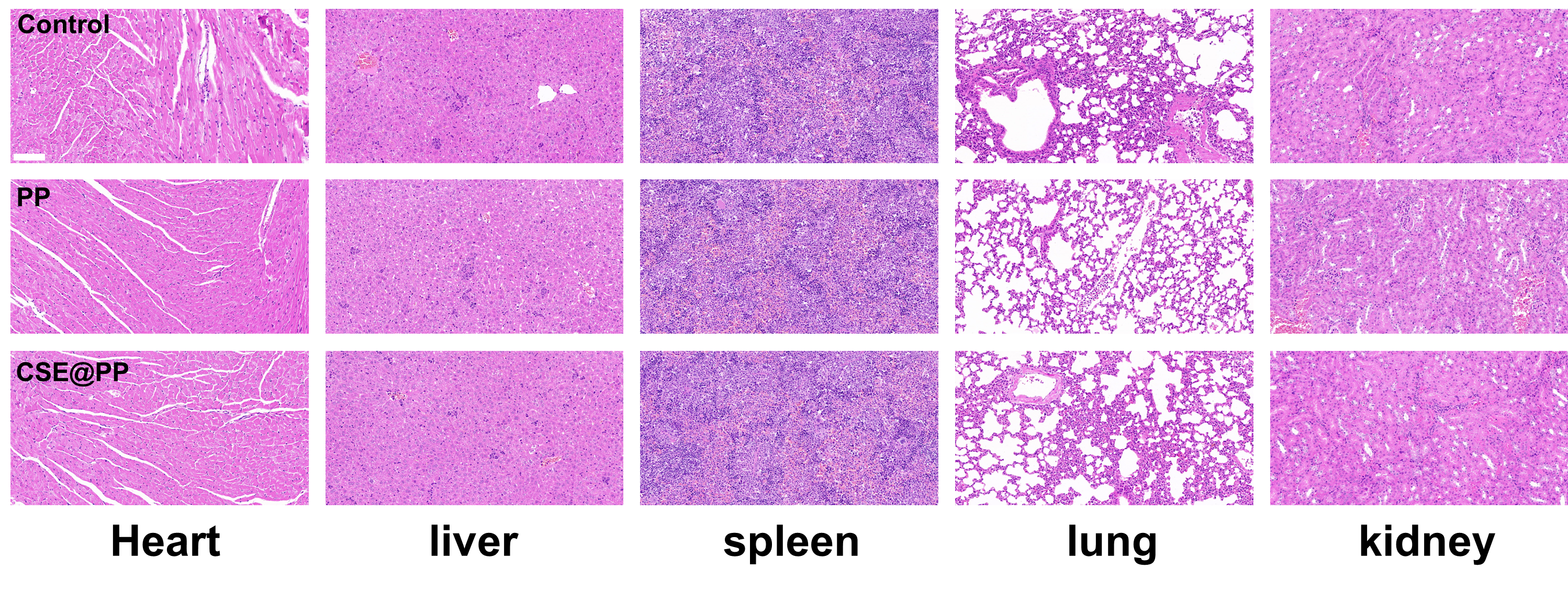


**Figure S16**. H&E staining in major organs (scale bar: 100 μm).


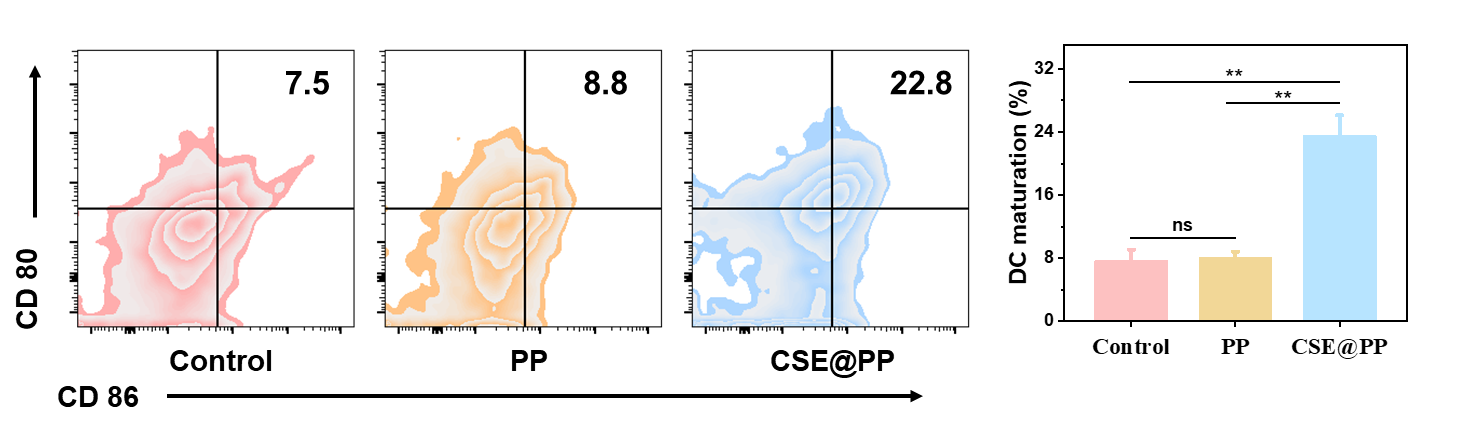


**Figure S17**. DC maturation in the tumor-draining lymph nodes of mice (n = 5 in each group; mean ± SD, **p < 0.01).


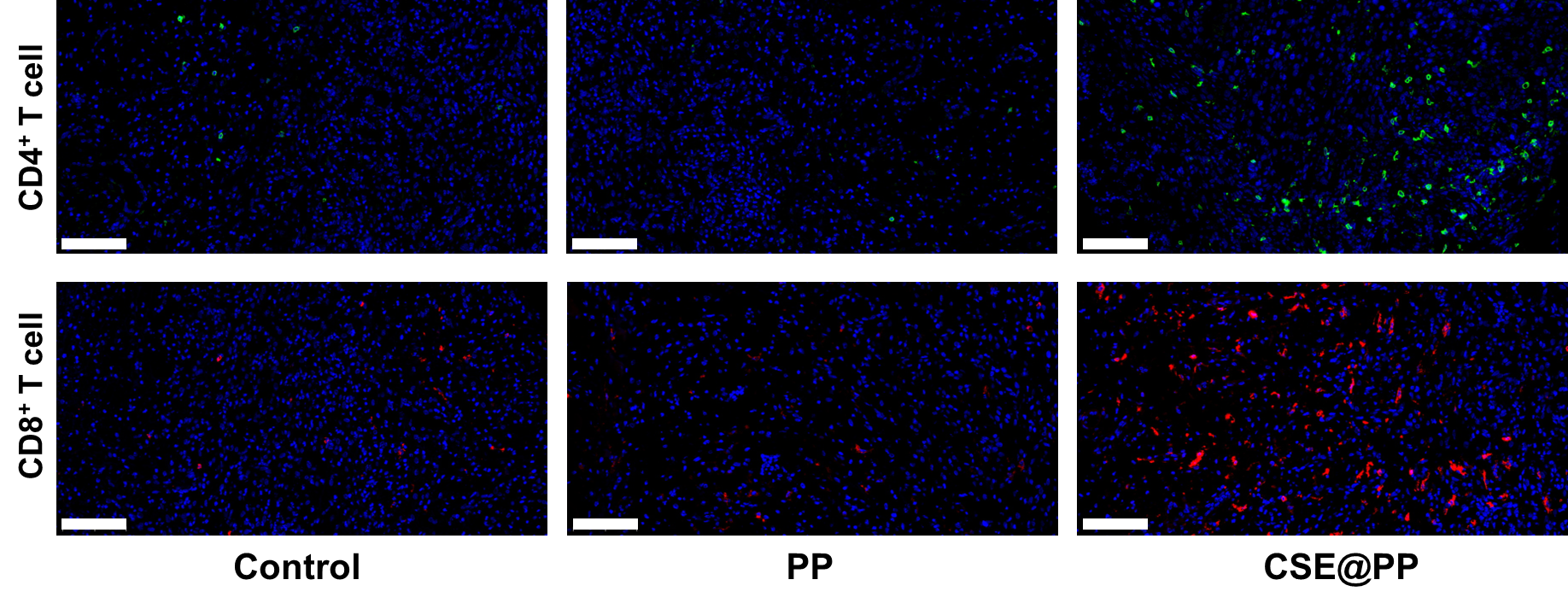


**Figure S18**. Histochemical immunofluorescence images of CD4^+^ and CD8^+^ T cells in the spleen of mice (scale bar: 100 μm).


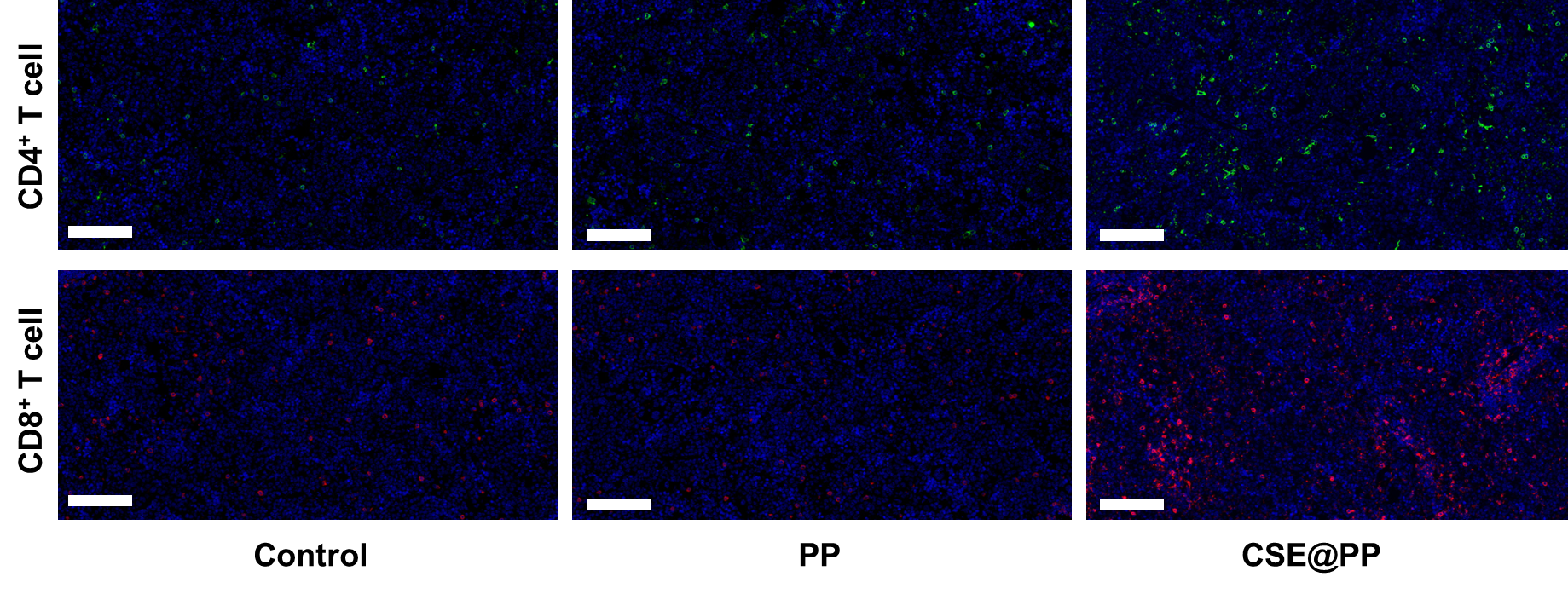


**Figure S19**. Histochemical immunofluorescence images of CD4^+^ and CD8^+^ T cells in the tumor of mice (scale bar: 100 μm).


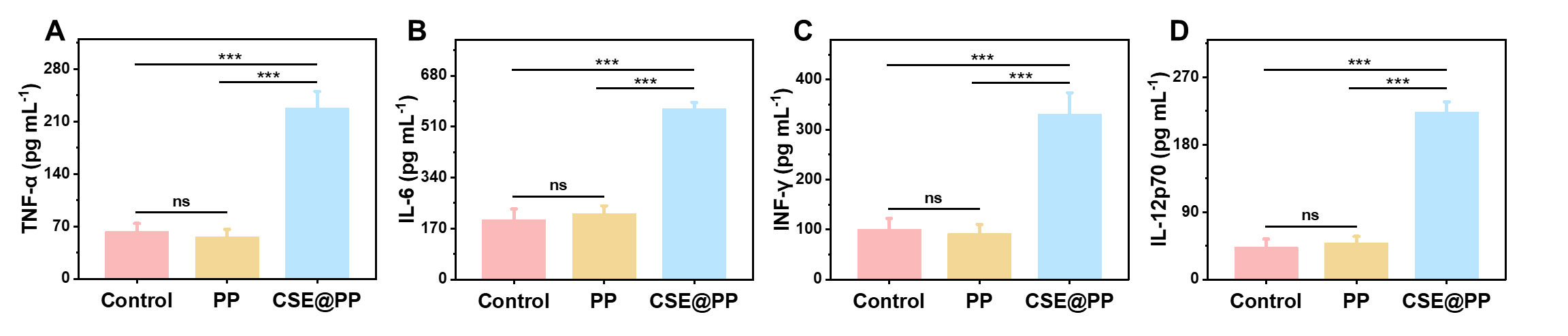


**Figure S20**. Secretion of cytokines in serum of mice. (A) TNF-α, (B) IL-6, (C) INF-γ, and (D) IL-12. (n = 5 in each group; mean ± SD, ***p < 0.001)


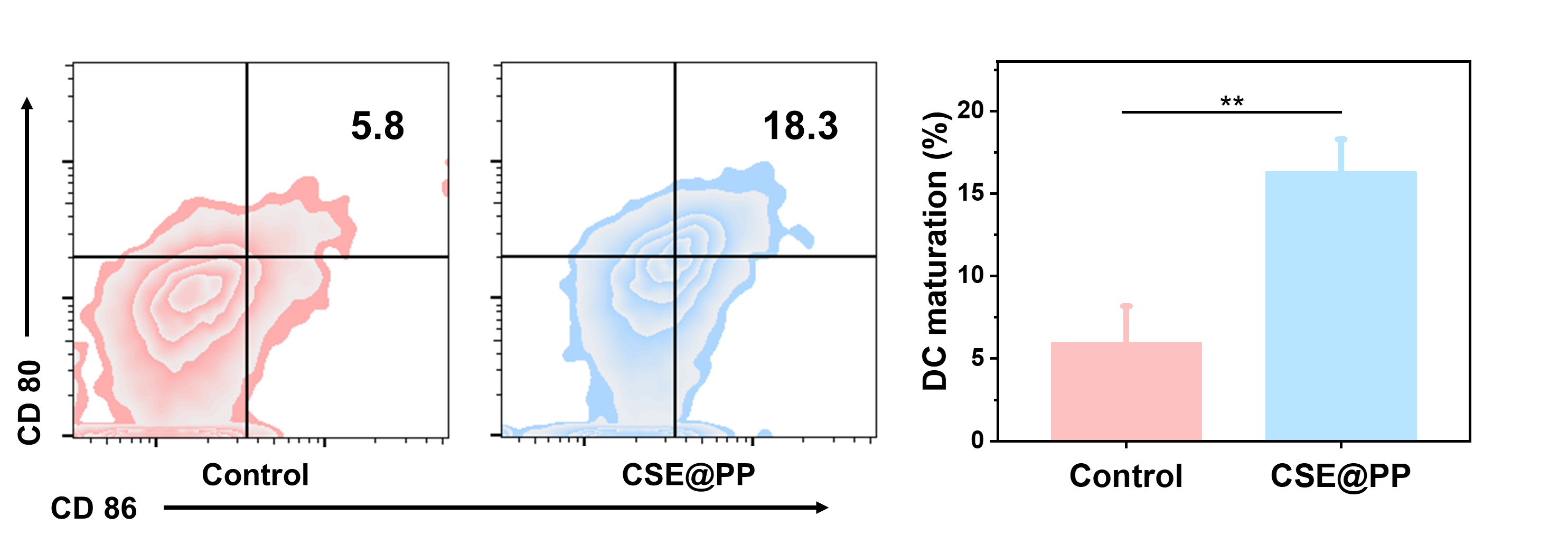


**Figure S21**. DC maturation in the distal tumor–draining lymph nodes of mice (n = 5 in each group; mean ± SD, **p < 0.01).


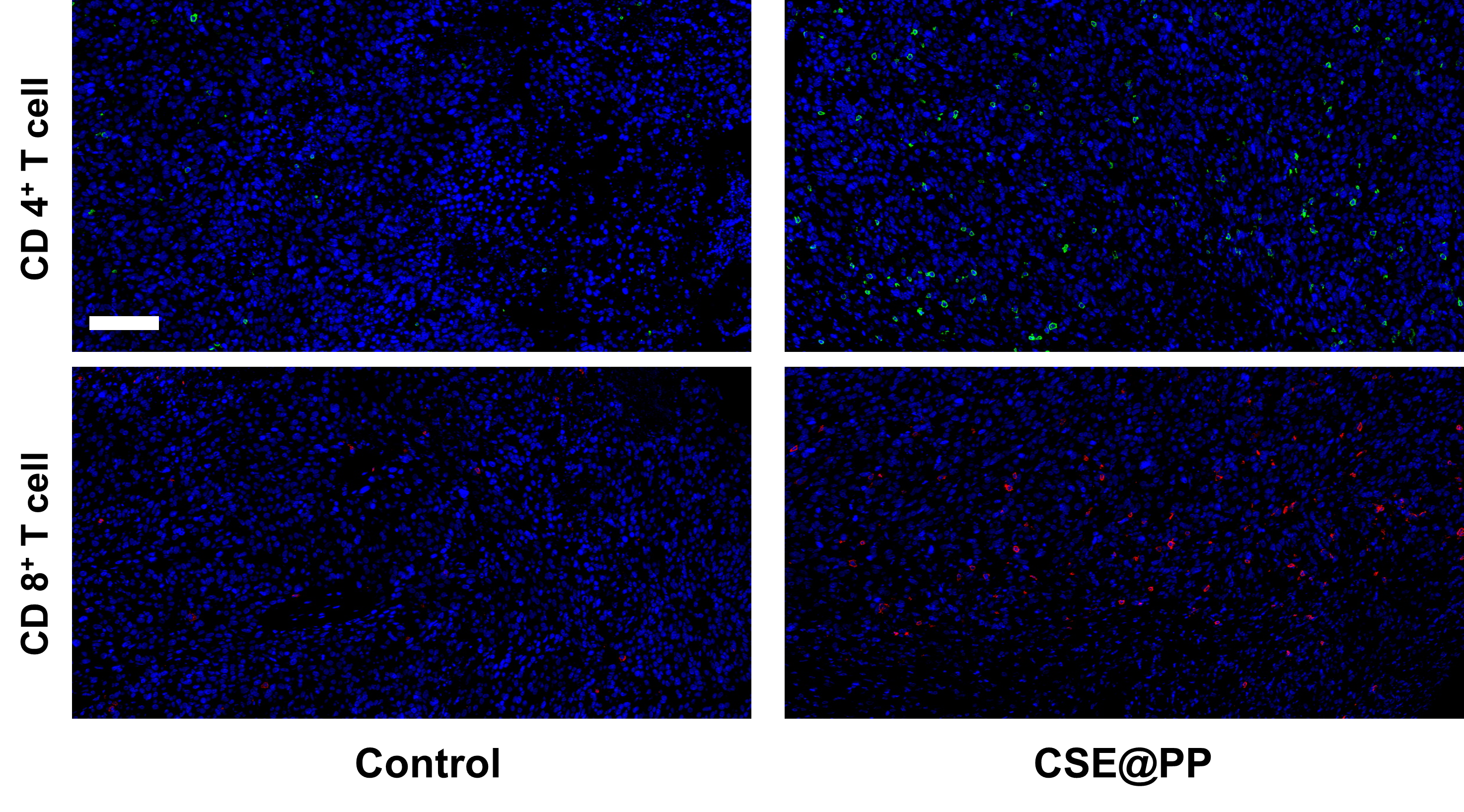


**Figure S22**. Histochemical immunofluorescence images of CD4^+^ and CD8^+^ T cells in in the tumor of mice (scale bar: 100 μm).
